# Health Benefits of Methylxanthines in Cacao and Chocolate

**DOI:** 10.3390/nu5104159

**Published:** 2013-10-18

**Authors:** Rafael Franco, Ainhoa Oñatibia-Astibia, Eva Martínez-Pinilla

**Affiliations:** 1 Cell and Molecular Neuropharmacology Laboratory, Neurosciences Division, Center for Applied Medical Research, Navarra University, Pamplona 31008, Spain; E-Mails: aonatibiaa@unav.es (A.O.); emartinezp@unav.es (E.M.); 2 Department of Biochemistry and Molecular Biology, University of Barcelona, Barcelona 08028, Spain

**Keywords:** adenosine, adenosine receptors, adenosine receptor antagonist, caffeine, theobromine

## Abstract

One may wonder why methylxanthines are so abundant in beverages used by humans for centuries, or in cola-drinks that have been heavily consumed since their appearance. It is likely that humans have stuck to any brew containing compounds with psychoactive properties, resulting in a better daily life, *i.e.*, more efficient thinking, exploring, hunting, *etc.*, however, without the serious side effects of drugs of abuse. The physiological effects of methylxanthines have been known for a long time and they are mainly mediated by the so-called adenosine receptors. Caffeine and theobromine are the most abundant methylxanthines in cacao and their physiological effects are notable. Their health-promoting benefits are so remarkable that chocolate is explored as a functional food. The consequences of adenosine receptor blockade by natural compounds present in cacao/chocolate are here reviewed. Palatability and health benefits of methylxanthines, in general, and theobromine, in particular, have further contributed to sustain one of the most innocuous and pleasant habits: chocolate consumption.

## 1. Introduction

**Figure 1:**
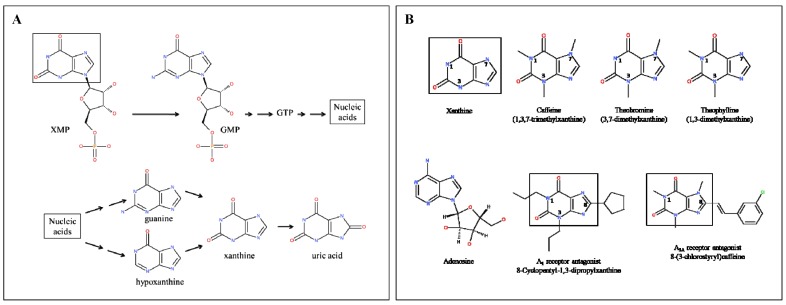
(**A**) Xanthine as an intermediate of GTP and nucleic acid anabolism and catabolism. Rectangles in compounds highlight the xanthine unit; (**B**) Structures of methylxanthines, adenosine, and adenosine receptor antagonists structurally related to methylxanthines. Rectangles in compounds highlight the xanthine unit.

Xanthines are a variety of compounds produced by plants and animals that have not been studied as frequently as other substances of metabolism, such as ATP or GTP. Interestingly, these compounds belong to the family of purines and all human cells produce them. In fact, xanthine and its derivatives are intermediates in the production of GMP, GDP, and GTP in cells that depend on a salvage pathway that recycles intermediates of degradation back into GTP and nucleic acids. In addition, xanthine has a role in the catabolism of nucleotides and nucleic acids, as it is the precursor of uric acid, which is the final product of the catabolism of the purines (Figure 1A). The anabolic and catabolic metabolism in which xanthine is involved overlap, *i.e.*, xanthine is at a crossroads from which GTP and nucleic acids may be synthesized by salvage mechanisms, or uric acid is produced to be eliminated in the urine. Interestingly, the benefits of xanthines in cacao are not at all related to the anabolic or catabolic purine metabolism in man. The active compounds in cacao that are structurally similar to xanthine are methylxanthines (Figure 1B), of which the action on human physiology is quite remarkable. Most consumers will be unaware that the psychoactive effects caused by cacao, coffee, or tea consumption results from blockade of adenosine receptors.

## 2. Flavonols and Methylxanthines in Cacao

Flavonols and methylxanthines are the most recognizable active components of cacao. Flavonols are polyphenolic structures that in cacao include catechin and derivatives, and B2, B3 and C1 procyanidins. Recent interest on these compounds derives from their antioxidant properties. Andújar *et al*. [1] have recently reviewed their potential benefits for human health. Among the myriad of health promoting effects suspected for antioxidants, anti-inflammatory actions seem to be promising. In fact, flavonols inhibit lipid peroxidation and affect production of lipid or lipid-derived molecules regulating the immune response and, recently, dietary cacao has been shown to ameliorate obesity-related inflammation in high fat-fed mice [2]. These molecules also seem to be key players in the increase of beneficial gut microbes (e.g., Lactobacilli) and the decrease of less beneficial ones (e.g., Clostridia) that follow cacao intake [3,4]. Hayek [5] has recently revisited data showing that cacao and/or chocolate modifies intestinal flora in the same way that prebiotics and probiotics do. As the functional interactions between gut microbiota and host metabolism are indeed shown in several studies (see for instance [6]), and result in health maintenance, some of the benefits of cacao may be due to this indirect mechanism.

Potential health-promoting benefits of flavonols in cacao require more experimental effort. Hence, this review is centered on methylxanthines that (i) are enriched in cacao and cacao-based products; (ii) have central actions because they readily cross the blood brain barrier; and (iii) have been heavily studied from a physiological and a clinical point of view.

## 3. Chocolate Appeal and Methylxanthine Reward

Historical and anthropological data demonstrate that man has searched for nutrients and/or beverages that contained substances that helped, not only calorically, but also in terms of well-being. Apart from cacao, coffee, and tea, other methylxanthine-rich beverages have been used in different cultures. For instance tejate has been ceremonially important but also as an essential staple, especially during periods of hard fieldwork; the beverage is a source of energy, fat, and methylxanthines [7]. Organic residue analysis of ceramics from Cahokia, the largest prehispanic site north of Mexico, reveals the use of an *Ilex*-derived black drink that contained theobromine, caffeine, and ursolic acid [8]. Indeed, it is quite likely that methylxanthines are at the root of the big interest of humans for certain types of plants.

Myths around the origins of coffee point to clear living benefits: (i) that of the lazy shepherd that begins to take good care of livestock after discovering and tasting coffee beans; and (ii) that of Omar, who, after being exiled to a desert discovered and enjoyed the decoctions obtained from roasted coffee beans. Implicitly these two stories take for granted the preservation of the habit of coffee consumption. Today, methylxanthines are still considered the main active components in cacao, coffee, and tea. Methylxanthines, acting on adenosine receptors in the central nervous system, enhance arousal, mood, and concentration levels [9], and make natural products containing methylxanthines to be explored as functional foods [10]. Drug reward in human evolution constitutes a paradox that has been recently detailed by Sullivan *et al*. [11]. Many of the drugs incorporated into human “diet”, from natural products, target neurons in the central nervous system and produce a variety of psychoactive, psychomotor, and neuroplastic effects whose description is out of the scope of the present review. However, apart from containing molecules acting on the central nervous system, successful beverages have appeal from a sensory perspective. In this aspect, methylxanthines appear as secondary, but important, players. Long time ago Schiffman *et al*. [12] reported that adaptation of the human tongue to methylxanthines potentiates taste. Concentrations of compounds used in the study were enough to block adenosine receptors, particularly the A_1_ subtype, while not affecting phosphodiesterase activity. The results were important to identify adenosine receptors as modulators of taste perception and to involve methylxanthines in the palatability of methylxanthine-containing foods. Although the molecular mechanism is unclear, the appeal of chocolate (and coffee and tea) to the sense of smell is generally known. A recent study has demonstrated that honeybees rewarded with caffeine, which occurs naturally in nectar of *Coffea* and *Citrus* species, were three times as likely to remember a learned floral scent as were honeybees rewarded with sucrose alone [13]. This effect is mediated by adenosine receptors in the Kenyon cells in mushroom bodies of the insect brain that are similar in function to hippocampal neurons. Although adenosine receptors are present in human hippocampus, the cognition-enhancing effects of caffeine in healthy subjects seem to be limited [9]. However as indicated below, sustained coffee consumption seems to be protective against suffering from Alzheimer’s disease.

## 4. Methylxanthine Levels

On the one hand, methylxanthines are plant-produced natural products. On the other hand, many of the plants used to prepare beverages for human consumption are enriched in methylxanthines. Linked to three of the most consumed beverages (coffee, tea, and cacao) are the most popular methylxanthines: caffeine, theophylline, and theobromine (Table 1). Caffeine is the most abundant methylxanthine in coffee, its level being smaller in chocolate than in coffee. Unlike coffee, chocolate is enriched in theobromine, and the level of theophylline is quite low in both cacao and coffee. Therefore this review will focus on the two main methylxanthines in cacao: caffeine and theobromine. Trognitz *et al*. [14] have recently reported the contents of caffeine and theobromine in unfermented and fermented cacao from 100 cacao trees (individual genotypes) representing groups of nine genotype spectra grown in Nicaragua. Theobromine concentration in fermented samples was always higher than that of caffeine although the theobromine/caffeine ratio was highly variable: 1.9 to 10.6. This variability surely impacts on the relative content of the two compounds in the final product, *i.e.*, in chocolate.

**Table 1:** Methylxanthine content in different cacao sources and products.

Source	Theobromine	Caffeine	Theophylline
Raw ground paste (mg/kg)	33,000	5600	200
Roasted ground paste (mg/kg)	36,000	330	Below limit of detection
Cacao ^a^ (mg/kg)	26,000	2400	Not determined
Cacao butter ^a^ (mg/kg)	140	400	Not determined
Cacao ^a^ (mg/kg)	4621	489	Below limit of detection
Baking chocolate ^a^ (mg/kg of sample)	10,040	1580	Below limit of detection
Milka chocolate ^a^ (mg/kg of sample)	1004	56	Below limit of detection
Dark chocolate ^b^ (mg/kg)	5000–7500	625–875	Not determined

Modified from Lo Coco *et al*. [15], ^a^ Srdjenovic *et al*. [16]; and/or Risner [17]; ^b^ Bruinsma and Taren [18]; The Hershey Company 2012; UK Joint Food Safety and Standards Group 1998.5. Safety of caffeine and theobromine.

Consumers intuitively notice that the effects of coffee and chocolate are not equal and the reason for that remains obscure. Different pharmacokinetics of the two main methylxanthines in cacao may be a relevant factor. Caffeine plasma concentration levels in coffee drinkers is 2–10 mg/L [19] and, although there is considerable interindividual variability, plasma clearance in 19–21 year-old male volunteers is higher for caffeine (2.1 (mL/min) × kg) than for theobromine (1.2 (mL/min) × kg). Consequently, the half-life in plasma is higher for theobromine (7.2 h) than for caffeine (4.1 h) [20]. The potency of different methylxanthines on different targets should also be taken into account. Whereas differences were not so marked in the case of adenosine receptors of the A_1_ subtype [21], the potency of blockade of methylxanthines in receptors of the A_2_ subtype (A_2A_ plus A_2B_) was more diverse. IC_50_ values were 45 and 98 µM for, respectively, theophylline and caffeine, and 2500 µM for theobromine. To our knowledge theobromine has not been tested on A_3_ receptors although the partner compounds (caffeine and theophylline) display very poor affinity for them [17]. Actual affinities of caffeine and theophylline for the four human adenosine receptor subtypes expressed in heterologous cells are in the range: 4–39 µM with the affinity of theophylline higher by a factor of two to four [22].

A variety of alternative and/or complementary targets have been attributed to methylxanthines. High doses of methylxanthines achieved by supplements may have a variety of actions acting on a variety of targets. However, at the blood levels found after beverage consumption, these alternative effects, for instance the recently reported interaction with DNA to modify its structure [23], are probably not relevant. Also, this review focuses more on the effects due to cacao/chocolate intake, and less on any potentially beneficial or detrimental effect of methylxanthines in diet supplements or over-the-counter drugs.

## 5. Safety of Caffeine and Theobromine

Theobromine is toxic for a variety of mammals such as dogs [24]. In contrast, the toxicity of methylxanthines in humans is very low. Overwhelming scientific evidence on caffeine proves that moderate consumption of caffeine from natural sources is safe [25,26]; theobromine seems to be even safer for humans (see below). The main pharmacological effects of methylxanthines include central nervous system stimulation, diuresis, cardiovascular and metabolic effects, bronchial relaxation and increased secretion of gastric acids [27]. Despite central actions, these substances do not produce chemical dependence. Even caffeine, which was under suspicion for years, was excluded in 2004 from the list of substances banned by the World Anti-Doping Agency (WADA). Previously, in 1958, the Food and Drug Administration (FDA) of the United States included caffeine within the list of “generally recognized as safe” substances, and considered in 1987 that an oral intake equivalent to 3–4 espresso cups (up to 300 mg/day) is safe for healthy adults. Caffeine is currently classified by the International Olympic Committee (IOC) as a substance restricted to 12 mg/L of urinary excretion, meaning that a dose of 300 mg (5 mg/kg for an athlete of 60 kg) would be within the limits accepted by the IOC. To our knowledge neither FDA, WADA, nor IOC has considered to put theobromine under close scrutiny. In both rodents and humans theobromine seems to be even safer than caffeine. Oral lethal dose 50 (DL_50_) for theobromine in rats is higher (1265 mg/kg) than for caffeine (192 mg/kg).

One of the advantages of natural compounds that are safe and do not produce chemical dependence is that they may be delivered to humans in clinical trials or in controlled studies in healthy volunteers. In a recent study in which different doses of theobromine (250 mg–1 g) were compared with a single dose of caffeine (200 mg), Baggott *et al*. [28] reported that theobromine at normal intake ranges may contribute to the positive effects of chocolate, but at higher intakes, effects may become negative. Apart from confirming safety at the highest dose even used in humans, the authors found that theobromine lacked caffeine-like self-reported effects. The benefits of chocolate on mood seem to be mainly exerted by caffeine whereas theobromine may be involved in qualitatively different actions. Toxic effects may appear upon consumption of diet supplements enriched in caffeine [29] or if caffeine is combined with drugs of abuse [30]. It should be also noted that the FDA announced in late 2010 that caffeine is unsafe as additive to alcoholic beverages [31].

## 6. Mechanism of Action of Methylxanthines

The first attempts to identify the potential targets for methylxanthines reported four different mechanisms. They were identified for caffeine acting on the central nervous system (reviewed [32]), but they may be extended to both other systems and other methylxanthines. They consist of: regulation of intracellular calcium level, phosphodiesterase inhibition, modulation of GABA_A_ receptor action, and antagonism of adenosine receptors. Caffeine and probably other methylxanthines stimulate calcium release from intracellular stores but at a high µM threshold concentration and with a maximal effect at 5–20 mM [33]. In addition, high concentrations are required for inhibition of phosphodiesterases [34], which are a family of enzymes that degrade cAMP and cGMP. It therefore seems that at the concentrations reached in dietary intake, these compounds are not effectively acting on phosphodiesterases or on calcium release. In addition, the low affinity of caffeine for GABA_A_ receptors [35] suggested that normal human caffeine consumption is unlikely to produce doses high enough to produce anxiogenic effects by inhibiting these receptors. In summary methylxanthines are mainly acting as adenosine receptor blockers, although other mechanisms may also operate, especially under the intake of caffeine supplements or methylxanthine-enriched medications.

## 7. Methylxanthines as Adenosine Receptor Antagonists

Quite interestingly the majority of receptor antagonists are synthetic, *i.e.*, developed in a laboratory, and a significant number of antagonist are used in therapy, for instance the so-called β-adrenergic-receptor blockers that are used in diseases of the cardiovascular system. Methylxanthines are natural products that behave as adenosine-receptor blockers and this is probably the reason cacao, coffee, and tea, have been so successful in the lifestyle of man. Four types of adenosine receptors have been identified that are widely distributed among human tissues. The widespread distribution of receptors fits with the presence of adenosine in every cell. In fact, adenosine exerts multiple actions in the central nervous, cardiovascular, *etc.*, systems that depend on the activation of adenosine receptors. They belong to the superfamily of G-protein-coupled receptors and may bind to Gs or to Gi, *i.e.*, activation of A_1_ and A_3_ subtypes leads to Gi-mediated decreases in cAMP and that of A_2A_ and A_2B_ leads to Gs-mediated increases in cAMP. The efficacy of blockers of A_2A_ receptors, which may be structurally similar to methylxanthines (Figure 1B), are under clinical evaluation for Parkinson’s disease, and they have been also proposed to combat one of the prevalent arrhythmias, atrial fibrillation, for which no drug is yet available [36,37]. When adenosine receptor antagonists are compared, theobromine appears as less potent than caffeine (see above), yet some of the actions displayed by cacao seem qualitatively different to those of coffee. Evidence by Ciruela *et al*. [38] suggested that caffeine may have different potencies depending on the cellular ambient in which adenosine receptors are placed, and on the capability of forming dimeric structures (adenosine receptor heterodimers). More recently, Orru *et al*. [39] have demonstrated that different adenosine receptor antagonists may have different potencies in the same receptor in different cells or in different locations in the cell. In neurons the effect of a given antagonist may markedly differ on targeting pre- *versus* post-synaptic adenosine receptors/receptor heterodimers. It is well known that the role of pre- is quite different from the role of post-synaptic receptors. This variable behavior of antagonists has, for instance, consequences in the effects of methylxanthines on motor control [39]. Therefore, a possibility that should be further explored is whether theobromine is preferentially acting on receptors, that on being blocked, lead to less unwanted effects than other methylxanthines such as caffeine or theophylline. This hypothesis would fully, or partly, explain why caffeine intake may lead to insomnia [40] whereas theobromine intake seems to favor sleep (see below).

## 8. Physiological and Health Benefits of Methylxanthines in Cacao

### 8.1. Theobromine in Oral Health

Benefits of theobromine have reached oral health and an interesting study made with extracted human third molars proved a consistent and remarkable protection of the enamel surface upon application of a 200 mg/L theobromine solution [41]. It should be noted that these high levels are not attained in natural sources but the results indeed open the way to consider supplementing toothpaste and/or mouthwash liquids with theobromine.

### 8.2. Methylxanthines in Respiratory Tract Diseases

Usmani *et al*. [42] were the first to describe that theobromine is able to suppress cough in both guinea-pigs and humans without the side effects displayed by other antitussive drugs, such as codeine. In the latter, a randomized, double-blind, placebo-controlled study showed that theobromine suppresses capsaicin-induced cough with no adverse effects. The actions of theobromine appear to be peripherally mediated, as it was directly affecting capsaicin-induced sensory nerve depolarization of guinea-pig and human vagus nerve, suggestive of an inhibitory effect on afferent nerve activation. At the molecular level, the antitussive effect may be due to blockade of adenosine receptors, which were detected in the guinea-pig trachea in 1980 by Coleman, to inhibition of phosphodiesterases, or both [43]. These and other complementary findings have prompted a variety of clinical trials. One trial (registered at US National Institutes of Health—NIH—with Reference No. NCT01416480), for which no results have been yet posted, was completed to assess in acute cough the efficacy of a combined treatment of theobromine and levodropropizine, which is acting in the periphery on an ill-defined target. Another trial on “study of the safety and effectiveness of theobromine capsules to treat frequent long-term cough” (NIH, Reference No. NCT01656668) is, as of May 2013, recruiting participants. Cough is a troubling symptom for some patients with cancer. Accordingly, Halfdanarson and Jatoi suggested, in 2007, a trial on dark chocolate as antitussive in patients with cancer; to our knowledge, such a study has not been undertaken [44]. Whereas being clinically evaluated in different countries by Texas-based Pernix™ and London-based Seek™, theobromine is a first-in-class non-codeine non-opioid drug for cough that has successfully completed trials and regulatory review in South Korea (sold as AnyCough™).

Epidemiological evidence suggests that theobromine and caffeine improve lung function and produce bronchodilatation in asthma patients [45,46]. Indeed, it has been demonstrated that patients with asthma and bronchitis may self-administer coffee or cacao/chocolate to relieve symptoms, even though the benefit may come from positive reinforcement [47].

A further beneficial effect of methylxanthines on the airways is related with the apnea of prematurity (AOP). AOP is a common problem affecting premature infants, likely secondary to an immature respiratory system. Methylxanthine therapy is a mainstay of treatment of central apnea by stimulating the central nervous system and respiratory muscle function [48]. Caffeine, as a non-selective antagonist of adenosine receptors, may improve minute ventilation, CO_2_ sensitivity, diaphragmatic contraction, respiratory muscle function, and neural respiratory drive, while decreasing the hypoxic depression of breathing [49,50].

### 8.3. Methylxanthines as Psycho-Stimulants

The psycho-stimulatory action of caffeine is well known [51] and, therefore, the caffeine in chocolate has a certain psycho-stimulant effect. The consensus related to a similar action of theobromine, which is the main methylxanthine component of cacao is in doubt. A careful study by Smit *et al*. [52] has demonstrated that the combination of caffeine and theobromine in the proportions found in cacao has psycho-stimulant effects. The authors performed two double-blind, placebo-controlled studies measuring the effects on cognitive performance and mood of the amounts of cacao powder and methylxanthines found in a 50 g bar of dark chocolate. In one of the studies voluntaries took visually identical portions of white chocolate, containing no methylxanthines, or low (8 mg caffeine + 100 mg theobromine) or high amounts (20 mg caffeine + 250 mg theobromine) of methyltxanthines. These three forms mimic, respectively, white, milk, and dark chocolate. Using a long duration simple reaction time task, a rapid visual information processing task, and a mood questionnaire, the results showed that the psycho-stimulant effect of chocolate is mainly due to the methylxanthines present [52].

### 8.4. Methylxanthines and Sleep

Grander *et al*. [53] have studied dietary nutrients associated with short and long sleep duration in a US nationally representative sample (*n* = 5587) showing that the largest contributor to sleep duration was theobromine. These results contrast with those known for caffeine, which causes insomnia in a percentage of the general population. It is not well-defined why some individuals become tolerant and may have good sleep even after intake of heavy caffeine loads coming from coffee or cola drinks. Apart from tolerance mechanisms, Yang *et al*. [54] have reviewed the literature to conclude that predisposition to caffeine use is highly specific to caffeine itself, and that genome association studies link polymorphisms in adenosine and dopamine receptors to caffeine-induced anxiety and sleep disturbances. The fact that cacao consumption is not linked to sleep disturbances and that theobromine is beneficial must be taken into appreciation.

### 8.5. Methylxanthines and Neurodegenerative Diseases

Despite coffee consumption was considered unsuitable for humans suffering a wide range of illnesses, it is nowadays considered a healthy habit (with few exceptions). As an illustrative example of the benefits of coffee consumption is a reduction in the incidence of two of the most prevalent neurodegenerative diseases: Parkinson’s [55] and Alzheimer’s [56,57]. The active component in actions on the central nervous system is assumed to be caffeine. Epidemiological studies, which are required to detect dietary styles that impact of the occurrence of a given disease, has to involve a high number of subjects and several years of duration. In the case of caffeine it seems that people that consume caffeinated coffee during the middle stages of life are less prone to suffer from neurological diseases when they get older. This hypothesis fits with the main role of methylxanthines, which is adenosine receptor blockade that in the brain results in higher neuronal activity thereby enabling a longer life for these cells. The higher neuronal activity may be due to a regulation in the perfusion of the brain [58,59,60] and/or an increase in cerebral oxygen consumption [61]. Another potential mechanism for neuroprotection may be an increased cerebrospinal fluid production [62,63].

### 8.6. Methylxanthines in Hypertension and Cardiovascular Diseases

Methylxanthines have a variety of effects in heart and in blood vessels. As early as in 1910, Bond *et al*. [64] reported “no change in the velocity of circulation” through the coronary arteries and veins by the action of caffeine or theobromine. Even before, in the XIX Century, Askanazi [65] introduced the continued administration of theobromine to prevent the attacks in angina pectoris. In a personal account of the experience with theobromine, Dock [66] indicated that “in most of the cases of angina no relief was given, but in an important minority relief was immediate and complete”. The patients who improved were usually those with frequent, sometimes very severe, pain, moderate sclerosis of palpable vessels, and no other demonstrable circulatory disease. At that time, the physician also wrote that “no patient with angina pectoris or intermittent claudication should be considered intractable or subject to operation until theobromine has been tried”. The treatment was defined as inexpensive and harmless, but its efficacy has been surpassed by more recent therapeutic approaches.

In the first half of the 20th Century, McGovern *et al*. [67] reported theobromine sodium salicylate as a vasodilator. Van den Bogaard *et al*. [68] have recently addressed the question of whether theobromine could be partially responsive of a blood pressure lowering effect. Consequently, a randomized, double-blind crossover trial with 41 subjects was undertaken to assess the effects of cacao with natural or high-dose theobromine on peripheral and central blood pressure. The two doses were carefully controlled, consisting of a natural dose of 106 mg of theobromine, or a theobromine-enriched cacao preparation with 979 mg of the compound. The results showed that a natural dose theobromine cacao did not significantly change either 24-h ambulatory or central systolic blood pressure compared with placebo. Furthermore, cacao drinks enriched with theobromine significantly increased 24-h ambulatory systolic blood pressure in a group of middle-aged subjects with high-normal blood pressure or grade I hypertension and low added risk of cardiovascular disease; despite the increased peripheral systolic blood pressure, central systolic blood pressure was lower two hours after consumption of theobromine-enriched cacao drinks. Whereas these data indicate that the cacao components (theobromine or else) at natural doses do not affect blood pressure in healthy and hypertension type I subjects, they may have a beneficial effect on other pathologies displaying hypertension [68]. In this sense, evidence from clinical studies has demonstrated that theobromine from cacao consumption significantly increases plasma HDL cholesterol levels, and decreases LDL concentration in plasma, conferring cardiovascular protection and reducing the risk of coronary heart disease [69,70]. On the other hand, the difference between central and peripheral blood pressure upon consumption of the theobromine-enriched beverages, is an important result that merits further investigation.

### 8.7. More Clinical Trials on Cacao Effects on Hypertension and Blood Vessel Status

It is worth noting that diverse clinical trials for which no results are yet posted have been filed to evaluate the effectiveness of cacao and/or its components on hypertension. One of the trials is addressed to examine the acute and chronic effect of consumption of flavanol-rich chocolate on endothelium function and blood pressure in healthy pregnant women (registered at NIH with Reference No. NCT01659060). The aim of the “Consumption of Chocolate in Pregnant Women”, CHOCENTA study (registered at NIH with Reference No. NCT01431443) that is recruiting participants in 2013, is to investigate the acute and chronic effect of consumption of flavanol-rich chocolate on endothelium function in pregnant women at high risk for preeclampsia, which is a high blood pressure condition after 20 weeks of pregnancy, in a woman who previously had normal blood pressure. Finally, there is another, somewhat similar to the reported by van den Bogaard *et al*. [68], registered at NIH with Reference No. NCT01672840 that pursues to know the effects of cacao on ambulatory blood pressure and vascular function in patients with stage I hypertension. In this particular “Dose And Response to Cacao” (DARC) study, which as of May 2013 is recruiting participants, the effects of daily consumption of two doses of cacao-containing products (dark chocolate plus cacao beverage) for eight weeks will be assayed by measuring 24-h diastolic blood pressure, endothelial function, and arterial stiffness.

### 8.8. Cacao in Insulin Resistance and Body Weight

In the absence of adenosine fat cell lipolysis may be stimulated by potent phosphodiesterase inhibitors; the ability of xanthines to stimulate basal and noradrenaline-stimulated lipolysis is however in agreement with their potency as adenosine antagonists [71]. Grassi *et al*. [72] reported that short-term administration of dark chocolate is followed by a significant increase in insulin sensitivity and a decrease in blood pressure in healthy persons. The effect was postulated to be due to flavonols in cacao although the involvement of adenosine receptor blockade cannot be ruled out [72,73,74]. It should be however noted that the benefits of the natural products in cacao may be offset by the heavy caloric intake due to chocolate consumption. The chocolate bars used in the Grassi *et al*. [72] study contained approximately 500 kcal, which roughly is 20%–25% of the recommended caloric intake per day. Unless sugar in cacao drinks or chocolate is totally or partially substituted by low caloric ingredients, the benefits of cacao on endocrine matters related to glucose and lipid handling can be contrasted, or even reversed, by increases in body weight [72,73].

## 9. Conclusions

Nowadays, apart from the benefits for day-life activities, methylxanthines may be even considered instrumental for health-maintenance. The combination of theobromine and caffeine in cacao/chocolate seems to be appropriate for having many of the expected benefits of methylxanthines with few drawbacks. In fact, chocolate/cacao does not generally produce insomnia or cause anxiety. The reason why the main methylxanthine in cacao, theobromine, has a differential effect when compared with caffeine or theophylline merits further investigation. Finally, it should be noted that the advantages of cacao/chocolate consumption may be counterbalanced by the high caloric intake associated to the sugar present in the preparations. Perhaps “diet” or “light” low-caloric preparations of cacao/chocolate should be explored to increase the health benefits of a pleasant experience.

## References

[1] Andújar I., Recio M.C., Giner R.M., Ríos J.L. (2012). Cocoa polyphenols and their potential benefits for human health. Oxid. Med. Cell. Longev..

[2] Gu Y., Yu S., Lambert J.D. (2013). Dietary cocoa ameliorates obesity-related inflammation in high fat-fed mice. Eur. J. Nutr..

[3] Redovniković I.R., Delonga K., Mazor S., Dragović-Uzelac V., Caric M., Vorkapic- Furac J. (2009). Polyphenolic content and composition, and antioxidative activity of different cocoa liquors. Czech J. Food Sci..

[4] Tzounis X., Rodriguez-Mateos A., Vulevic J., Gibson G.R., Kwik-Uribe C., Spencer J.P. (2011). Prebiotic evaluation of cocoa-derived flavanols in healthy humans by using a randomized, controlled, double-blind, crossover intervention study. Am. J. Clin. Nutr..

[5] Hayek N. (2013). Chocolate, gut microbiota, and human health. Front. Pharmacol..

[6] Tremaroli V., Bäckhed F. (2012). Functional interactions between the gut microbiota and host metabolism. Nature.

[7] Sotelo A., Soleri D., Wacher C., Sánchez-Chinchillas A., Argote R.M. (2012). Chemical and nutritional composition of tejate, a traditional maize and cacao beverage from the Central Valleys of Oaxaca, Mexico. Plant Foods Hum. Nutr..

[8] Crown P.L., Emerson T.E., Gu J., Hurst W.J., Pauketat T.R., Ward T. (2012). Ritual black drink consumption at Cahokia. Proc. Natl. Acad. Sci. USA.

[9] Nehlig A. (2010). Is caffeine a cognitive enhancer?. J. Alzheimer’s Dis..

[10] Dórea J.G., da Costa T.H. (2005). Is coffee a functional food?. Br. J. Nutr..

[11] Sullivan R.J., Hagen E.H., Hammerstein P. (2008). Revealing the paradox of drug reward in human evolution. Proc. Biol. Sci..

[12] Schiffman S.S., Gill J.M., Diaz C. (1985). Methylxanthines enhance taste: Evidence for modulation of taste by adenosine receptor. Pharmacol. Biochem. Behav..

[13] Wright G.A., Baker D.D., Palmer M.J., Stabler D., Mustard J.A., Power E.F., Borland A.M., Stevenson P.C. (2013). Caffeine in floral nectar enhances a pollinator’s memory of reward. Science.

[14] Trognitz B., Cros E., Assemat S., Davrieux F., Forestier-Chiron N., Ayestas E., Kuant A., Scheldeman X., Hermann M. (2013). Diversity of cacao trees in Waslala, Nicaragua: Associations between genotype spectra, product quality and yield potential. PLoS One.

[15] Lo Coco F., Lanuzza F., Micali G., Cappellano G. (2007). Determination of theobromine, theophylline, and caffeine in by-products of cupuacu and cacao seeds by high-performance liquid chromatography. J. Chromatogr. Sci..

[16] Srdjenovic B., Djordjevic-Milic V., Grujic N., Injac R., Lepojevic Z. (2008). Simultaneous HPLC determination of caffeine, theobromine, and theophylline in food, drinks, and herbal produts. J. Chromatogr. Sci..

[17] Risner C.H. (2008). Simultaneous determination of theobromine, (+)-catechin, caffeine, and (−)-epicatechin in standard reference material baking chocolate 2384, cocoa, cocoa beans, and cocoa butter. J. Chromatogr. Sci..

[18] Bruinsma K., Taren D.L. (1999). Chocolate: Food or drug?. J. Am. Diet. Assoc..

[19] Baselt R.C. (1982). Disposition of Toxic Drugs and Chemicals in Man.

[20] Lelo A., Birkett D.J., Robson R.A., Miners J.O. (1986). Comparative pharmacokinetics of caffeine and its primary demethylated metabolites paraxanthine, theobromine and theophylline in man. Br. J. Clin. Pharmacol..

[21] Fredholm B.B., Persson C.G. (1982). Xanthine derivatives as adenosine receptor antagonists. Eur. J. Pharmacol..

[22] Fredholm B.B., Irenius E., Kull B., Schulte G. (2001). Comparison of the potency of adenosine as an agonist at human adenosine receptors expressed in Chinese hamster ovary cells. Biochem. Pharmacol..

[23] Johnson I.M., Prakash H., Prathiba J., Raghunathan R., Malathi R. (2012). Spectral analysis of naturally occurring methylxanthines (theophylline, theobromine and caffeine) binding with DNA. PLoS One.

[24] Gans J.H., Korson R., Cater M.R., Ackerly C.C. (1980). Effects of short-term and long-term theobromine administration to male dogs. Toxicol. Appl. Pharmacol..

[25] Clark N. (1997). Caffeine: A user’s guide. Phys. Sports Med..

[26] Higdon J.V., Frei B. (2006). Coffee and health: A review of recent human research. Crit. Rev. Food Sci. Nutr..

[27] Hornfeldt C.S. (1987). Chocolate toxicity in dogs. Mod. Vet. Pract..

[28] Baggott M.J., Childs E., Hart A.B., de Bruin E., Palmer A.A., Wilkinson J.E., de Wit H. (2013). Psychopharmacology of theobromine in healthy volunteers. Psychopharmacology.

[29] Pendleton M., Brown S., Thomas C., Odle B. (2012). Potential toxicity of caffeine when used as a dietary supplement for weight loss. J. Diet. Suppl..

[30] Sinchai T., Plasen S., Sanvarinda Y., Jaisin Y., Govitrapong P., Morales N.P., Ratanachamnong P., Plasen D. (2011). Caffeine potentiates methamphetamine-induced toxicity both *in vitro* and *in vivo*. Neurosci. Lett..

[31] Arria A.M., O’Brien M.C. (2011). The “high” risk of energy drinks. JAMA.

[32] Chen J.F., Chern Y. (2011). Impacts of methylxanthines and adenosine receptors on neurodegeneration: Human and experimental studies. Handb. Exp. Pharmacol..

[33] McPherson P.S., Kim Y.K., Valdivia H., Knudson C.M., Takekura H., Franzini-Armstrong C., Coronado R., Campbell K.P. (1991). The brain ryanodine receptor: A caffeine-sensitive calcium release channel. Neuron.

[34] Choi O.H., Shamim M.T., Padgett W.L., Daly J.W. (1988). Caffeine and theophylline analogues: correlation of behavioral effects with activity as adenosine receptor antagonists and as phosphodiesterase inhibitors. Life Sci..

[35] Marangos P.J., Paul S.M., Parma A.M., Goodwin F.K., Syapin P., Skolnick P. (1979). Purinergic inhibition of diazepam binding to rat brain (*in vitro*). Life Sci..

[36] Hove-Madsen L., Prat-Vidal C., Llach A., Ciruela F., Casadó V., Lluis C., Bayes-Genis A., Cinca J., Franco R. (2006). Adenosine A2A receptors are expressed in human atrial myocytes and modulate spontaneous sarcoplasmic reticulum calcium release. Cardiovasc. Res..

[37] Llach A., Molina C.E., Prat-Vidal C., Fernandes J., Casadó V., Ciruela F., Lluís C., Franco R., Cinca J., Hove-Madsen L. (2011). Abnormal calcium handling in atrial fibrillation is linked to up-regulation of adenosine A2A receptors. Eur. Heart J..

[38] Ciruela F., Casadó V., Rodrigues R.J., Luján R., Burgueño J., Canals M., Borycz J., Rebola N., Goldberg S.R., Mallol J. (2006). Presynaptic control of striatal glutamatergic neurotransmission by adenosine A1-A2A receptor heteromers. J. Neurosci..

[39] Orru M., Bakešová J., Brugarolas M., Quiroz C., Beaumont V., Goldberg S.R., Lluís C., Cortés A., Franco R., Casadó V. (2011). Striatal pre- and postsynaptic profile of adenosine A2A receptor antagonists. PLoS One.

[40] Lazarus M., Shen H.Y., Cherasse Y., Qu W.M., Huang Z.L., Bass C.E., Winsky-Sommerer R., Semba K., Fredholm B.B., Boison D. (2011). Arousal effect of caffeine depends on adenosine A_2A_ receptors in the shell of the nucleus accumbens. J. Neurosci..

[41] Kargul B., Ozcan M., Peker S., Nakamoto T., Simmons W.B., Falster A.U. (2012). Evaluation of human enamel surfaces treated with theobromine: A pilot study. Oral Health Prev. Dent..

[42] Usmani O.S., Belvisi M.G., Patel H.J., Crispino N., Birrell M.A., Korbonits M., Korbonits D., Barnes P.J. (2005). Theobromine inhibits sensory nerve activation and cough. FASEB J..

[43] Coleman R.A. (1980). Purine antagonists in the identification of adenosine-receptors in guinea-pig trachea and the role of purines in non-adrenergic inhibitory neurotransmission. Br. J. Pharmacol..

[44] Halfdanarson T.R., Jatoi A. (2007). Chocolate as a cough suppressant: Rationale and justification for an upcoming clinical trial. Support. Cancer Ther..

[45] Bara A.I., Barley E.A. (2001). Caffeine for asthma. Cochrane Database Syst. Rev..

[46] Simons F.E., Becker A.B., Simons K.J., Gillespie C.A. (1985). The bronchodilator effect and pharmacokinetics of theobromine in young patients with asthma. J. Allergy Clin. Immunol..

[47] Pagano R., Negri E., Decarli A., La Vecchia C. (1988). Coffee drinking and prevalence of bronchial asthma. Chest.

[48] Zhao J., Gonzalez F., Mu D. (2011). Apnea of prematurity: From cause to treatment. Eur. J. Pediatr..

[49] Aranda J.V., Beharry K., Valencia G.B., Natarajan G., Davis J. (2010). Caffeine impact on neonatal morbidities. J. Matern. Fetal Neonatal Med..

[50] Henderson-Smart D.J., Steer P.A. (2010). Caffeine *versus* theophylline for apnea in preterm infants. Cochrane Database Syst. Rev..

[51] Franco R. (2009). Coffee and mental health. Aten. Primaria.

[52] Smit H.J., Gaffan E.A., Rogers P.J. (2004). Methylxanthines are the psycho-pharmacologically active constituents of chocolate. Psychopharmacology.

[53] Grandner M.A., Jackson N., Gerstner J.R., Knutson K.L. (2013). Dietary nutrients associated with short and long sleep duration. Data from a nationally representative sample. Appetite.

[54] Yang A., Palmer A.A., de Wit H. (2010). Genetics of caffeine consumption and responses to caffeine. Psychopharmacology.

[55] Costa J., Lunet N., Santos C., Santos J., Vaz-Carneiro A. (2010). Caffeine exposure and the risk of Parkinson’s disease: A systematic review and meta-analysis of observational studies. J. Alzheimer’s Dis..

[56] Maia L., de Mendonca A. (2002). Does caffeine intake protect from Alzheimer’s disease?. Eur. J. Neurol..

[57] Eskelinen M.H., Ngandu T., Tuomilehto J., Soininen H., Kivipelto M. (2009). Midlife coffee and tea drinking and the risk of late-life dementia: A population-based CAIDE study. J. Alzheimer’sDis..

[58] Pelligrino D.A., Xu H.L., Vetri F. (2010). Caffeine and the control of cerebral hemodynamics. J. Alzheimer’s Dis..

[59] Klaassen E.B., de Groot R.H., Evers E.A., Snel J., Veerman E.C., Ligtenberg A.J., Jolles J., Veltman D.J. (2013). The effect of caffeine on working memory load-related brain activation in middle-aged males. Neuropharmacology.

[60] Koppelstaetter F., Poeppel T.D., Siedentopf C.M., Ischebeck A., Verius M., Haala I., Mottaghy F.M., Rhomberg P., Golaszewski S., Gotwald T. (2008). Does caffeine modulate verbal working memory processes? An fMRI study. Neuroimage.

[61] Haller S., Rodriguez C., Moser D., Toma S., Hofmeister J., Sinanaj I., Van De Ville D., Giannakopoulos P., Lovblad K.O. (2013). Acute caffeine administration impact on working memory-related brain activation and functional connectivity in the elderly: A BOLD and perfusion MRI study. Neuroscience.

[62] Han M.E., Kim H.J., Lee Y.S., Kim D.H., Choi J.T., Pan C.S., Yoon S., Baek S.Y., Kim B.S., Kim J.B. (2009). Regulation of cerebrospinal fluid production by caffeine consumption. BMC Neuroscience.

[63] Wostyn P., van Dam D., Audenaert K., de Deyn P.P. (2011). Increased cerebrospinal fluid production as a possible mechanism underlying Caffeine’s protective effect against Alzheimer’s disease. Int. J. Alzheimer’s Dis..

[64] Bond G.S. (1910). Effect of various agents on the blood flow through the coronary arteries and veins. J. Exp. Med..

[65] Askanazy S. (1986). Klinisches über Diuretin. Deutsches Archiv für klinische Medicin.

[66] Dock W. (1926). The use of theobromine for pain of arteriosclerotic origin. Calif. West. Med..

[67] McGovern T., McDevitt E., Wright I.S. (1936). Theobromine sodium salicylate as a vasodilator. J. Clin. Investig..

[68] Van den Bogaard B., Draijer R., Westerhof B.E., van den Meiracker A.H., van Montfrans G.A., van den Born B.J. (2010). Effects on peripheral and central blood pressure of cocoa with natural or high-dose theobromine: A randomized, double-blind crossover trial. Hypertension.

[69] Khan N., Monagas M., Andres-Lacueva C., Casas R., Urpí-Sardà M., Lamuela-Raventós R.M., Estruch R. (2012). Regular consumption of cocoa powder with milk increases HDL cholesterol and reduces oxidized LDL levels in subjects at high-risk of cardiovascular disease. Nutr. Metab. Cardiovasc. Dis..

[70] Neufingerl N., Zebregs Y.E., Schuring E.A., Trautwein E.A. (2013). Effect of cocoa and theobromine consumption on serum HDL-cholesterol concentrations: A randomized controlled trial. Am. J. Clin. Nutr..

[71] Fredholm B.B., Lindgren E. (1984). The effect of alkylxanthines and other phosphodiesterase inhibitors on adenosine-receptor mediated decrease in lipolysis and cyclic AMP accumulation in rat fat cells. Acta Pharmacol. Toxicol..

[72] Grassi D., Lippi C., Necozione S., Desideri G., Ferri C. (2005). Short-term administration of dark chocolate is followed by a significant increase in insulin sensitivity and a decrease in blood pressure in healthy persons. Am. J. Clin. Nutr..

[73] Kelly C.J. (2005). Effects of theobromine should be considered in future studies. Am. J. Clin. Nutr..

[74] Figler R.A., Wang G., Srinivasan S., Jung D.Y., Zhang Z., Pankow J.S., Ravid K., Fredholm B., Hedrick C.C., Rich S.S. (2011). Links between insulin resistance, adenosine A_2B_ receptors, and inflammatory markers in mice and humans. Diabetes.

